# Melioidosis in Tsunami Survivors

**DOI:** 10.3201/eid1110.050740

**Published:** 2005-10

**Authors:** Eugene Athan, Anthony M. Allworth, Catherine Engler, Ivan Bastian, Allen C. Cheng

**Affiliations:** * The Geelong Hospital, Geelong, Victoria, Australia; † Royal Brisbane Hospital, Brisbane, Queensland, Australia; ‡ Queensland Health Pathology Services, Townsville, Queensland, Australia; § Institute of Medical and Veterinary Science, Adelaide, South Australia, Australia; ¶ Menzies School of Health Research, Darwin, Northern Territory, Australia

**Keywords:** tsunami, pneumonia, melioidosis, letter

**To the Editor:** A tsunami devastated coastal areas of the Indian Ocean rim in December 2004. Of the affected countries, more than half of the ≈300,000 deaths occurred in the Aceh Province of Indonesia, close to the epicenter of the earthquake near northern Sumatra. Infrastructure, including medical and laboratory facilities, in this region was severely damaged. Of >1,000,000 survivors, >500,000 likely were injured. Most injuries were from trauma, but a substantial number were caused by aspiration of, or immersion in, saltwater that may have been contaminated by soil, sewage, or other environmental sources.

Melioidosis, caused by the saprophytic gram-negative bacillus *Burkholderia pseudomallei*, is endemic in Southeast Asia and northern Australia. Most cases have been found in northeastern Thailand, Singapore, and northern Australia. Melioidosis has been reported only sporadically from Indonesia and mainly in returning travelers (*1**–**3*).

In the context of acute medical relief efforts to the town of Banda Aceh, we report on 10 patients with pneumonia, including 4 patients with culture-confirmed melioidosis, after their immersion in contaminated saltwater during the tsunami. Clinical and laboratory services were reestablished on January 3, 2005, at Fakinah Hospital and on January 13, 2005, at Zainoel Abidin Hospital by the relief teams. Patients were identified opportunistically; details of treatment and outcome were reviewed retrospectively. Cultures were taken when clinically indicated and when specimens were available. Sputum cultures were plated onto horse blood agar, cystine lactose electrolyte-deficient agar, *Haemophilus* agar, and colistin/nalidixic acid blood agar incubated in a candle jar at 35°C for <3 days. Colonies suspicious for *B. pseudomallei* were subcultured to *B. cepacia*–selective media (Oxoid, Adelaide, South Australia, Australia). Blood cultures and screening cultures of throat and rectum specimens were not performed routinely. Isolates of *B. pseudomallei* and characteristic antimicrobial drug susceptibilities were confirmed with API20NE (bioMérieux, Marcy l'Etoile, France).

From January 3 to January 28, 2005, a total of 10 cases of postimmersion pneumonia were identified. All patients were <18 years of age and previously well; 6 were male. No cases were epidemiologically linked to others. The patients were treated 10–35 days after the tsunami. Eight had bilateral alveolar opacities on chest radiograph; 3 also had empyema. Clinical, radiologic, and microbiologic details are summarized in the Table.

**Table:** Clinical details of pneumonia patients after tsunami in Banda Aceh, Indonesia, January 2003*

Patient no.	Age (y)/sex	Radiograph results	Complications	Microbiologic test results	Days before treatment	Antimicrobial drug	Acute outcome
1	7/M	Bilateral infiltrates	Cavitation	No cultures taken	19	Meropenem	Fever resolved, improving @30 days
2	12/M	Bilateral infiltrates		*Pseudomonas aeruginosa* (sputum)	37	Meropenem	Serious but stable @12 days
3	5/M	Unilateral infiltrates	Cavitation	No cultures taken	10	Meropenem	Fever resolved, improving @39 days
4	15/F	Bilateral infiltrates	Pneumothorax, empyema	*Burkholderia pseudomallei* (sputum)	27	Meropenem 2 wks, then oral TMP-SMX and coamoxiclav	Fully recovered, discharged @20 days
5	14/M	Bilateral infiltrates		*P. aeruginosa* (sputum)	35	Meropenem	Improving @11 days
6	6/M	Bilateral infiltrates		*P. aeruginosa* (sputum)	30	Meropenem	Improving @19 days
7	18 mo/M	Bilateral infiltrates	Empyema	*B. pseudomallei* (pleural fluid)	30	Meropenem	Improving @23 days
8	10/F	Bilateral infiltrates	Empyema	*P. aeruginosa* *B. pseudomallei* *Klebsiella* sp. (pleural fluid)	30	Meropenem	Improving @23 days
9	13/F	Bilateral infiltrates		*P. aeruginosa* *B. pseudomallei* (sputum)	33	Meropenem	Improving @18 days
10	17/F	Unknown	Unknown	*Nocardia* spp. (sputum)	21	Ticarcillin/clavulanate, ciprofloxacin	Not improving, outcome unknown @12 days

*ICU, intensive care unit; TMP-SMX, trimethoprim-sulfamethoxazole.

The sputum cultures of 4 patients were positive for *B. pseudomallei*. Except posttsunami exposure, none had risk factors for melioidosis, including diabetes, renal failure, or thalassemia. Other co-isolated organisms included *Pseudomonas aeruginosa* and *Klebsiella* sp.; 2 patients who did not have cultures taken had cavitatory lung disease. All patients with melioidosis were treated with meropenem, and all but 1 clinically improved in the hospital.

This is the second report of melioidosis from within Indonesia (*1*) and the second published report of melioidosis after the tsunami disaster (*4*). Cases from this event were included in a preliminary communication (*5*). However, exported cases of melioidosis from Indonesia, as well as the neighboring countries of Singapore and Malaysia, have been reported previously (*2**,**3*), a likely indication that this infection is underrecognized in Indonesia. Melioidosis has also been reported in tsunami survivors from Sri Lanka (*4*) and Thailand.

A striking feature of this event is the lack of predisposing factors and the young age of the patients. This feature likely represents the unique mode of acquisition and magnitude of the infecting inoculum, as well as the vulnerability of children to near drowning after flooding. One third of the patients had empyema, which reflects both the severity of pulmonary disease and delay in receiving medical care. Undoubtedly, a substantial selection bias occurred by including only patients who sought hospital treatment, and this is suggested by the low death rate. Melioidosis, acquired after near drowning, has been associated with a short incubation period and severe disease. However, patients who had melioidosis before medical aid arrived in the region would likely have died. Patients with longer incubation periods also may have acquired melioidosis through contaminated wounds with subsequent hematogenous dissemination.

Medical services to the region were by no means comprehensive during this time. Many other patients with postimmersion pneumonia and melioidosis may have been overlooked, both during the study (for persons who were not treated or if cultures were not taken) and afterwards; the incubation period of melioidosis may be <62 years. A further limitation is the lack of denominator data because no reliable records were kept on hospital admissions, and the exact number of survivors is not yet known. Conflicting data are found on the accuracy of the API20NE test kit used to identify bacteria in this study (*6**–**9*), but we believe that the clinical features and microbiologic findings suggest melioidosis.

This report confirms that *B. pseudomallei* exists in the Aceh Province of Indonesia and that melioidosis and gram-negative pneumonia may complicate saltwater immersion in this region. After near drowning incidents, melioidosis is characterized by severe pulmonary disease, including pleural effusions. Clinicians worldwide should be mindful that melioidosis in tsunami survivors may appear many years after exposure.
